# Mathematical modeling of hypoxia and adenosine to explore tumor escape mechanisms in DC-based immunotherapy

**DOI:** 10.1038/s41598-024-62209-6

**Published:** 2024-05-18

**Authors:** Elahe Ghiyabi, Abazar Arabameri, Mostafa Charmi

**Affiliations:** Department of Electrical Engineering, University of Zanjan, Zanjan, Iran

**Keywords:** Tumor escape mechanisms, Hypoxia, Adenosine, Dendritic cell-based immunotherapy, Mathematical modeling, Cancer, Computational biology and bioinformatics, Immunology, Systems biology

## Abstract

Identifying and controlling tumor escape mechanisms is crucial for improving cancer treatment effectiveness. Experimental studies reveal tumor hypoxia and adenosine as significant contributors to such mechanisms. Hypoxia exacerbates adenosine levels in the tumor microenvironment. Combining inhibition of these factors with dendritic cell (DC)-based immunotherapy promises improved clinical outcomes. However, challenges include understanding dynamics, optimal vaccine dosages, and timing. Mathematical models, including agent-based, diffusion, and ordinary differential equations, address these challenges. Here, we employ these models for the first time to elucidate how hypoxia and adenosine facilitate tumor escape in DC-based immunotherapy. After parameter estimation using experimental data, we optimize vaccination protocols to minimize tumor growth. Sensitivity analysis highlights adenosine’s significant impact on immunotherapy efficacy. Its suppressive role impedes treatment success, but inhibiting adenosine could enhance therapy, as suggested by the model. Our findings shed light on hypoxia and adenosine-mediated tumor escape mechanisms, informing future treatment strategies. Additionally, identifiability analysis confirms accurate parameter determination using experimental data.

## Introduction

Cancer is one of the most frequent causes of mortality globally^1,2^. Hypoxia and adenosine are two of the factors that compromise the immune system and contribute to the spread of cancer^3,4^.

Uncontrolled cell growth in the tumor microenvironment (TME) frequently exceeds the capacity of the preexisting blood capillaries to supply oxygen demand, resulting in hypoxic conditions^5^. Hypoxia has been demonstrated to protect tumors by promoting angiogenesis, reducing antitumor immune cells, and activating immunosuppressive cells like regulatory T cells (Tregs)^6^. Inhibiting the transcription factors known as hypoxia-inducing factors (HIFs), which react to reductions in the amount of available oxygen in the cellular environment, can be a useful strategy for limiting tumor growth^4,7,8^.

The ectoenzymes such as CD73 generate extracellular adenosine from ATP or ADP, which has a significant impact on the activation of immune system suppressor cells like Tregs, as well as tumor angiogenesis and metastasis^9^. So, CD73 is an attractive therapeutic target for the treatment of cancer, particularly when used in conjunction with conventional therapy (chemotherapy, radiotherapy)^3,10–12^. On the other hand, the relationship between adenosine and hypoxia has also been investigated in experimental studies and it has been shown that CD73 increases with decreasing oxygen levels^13–16^.

In dendritic cell (DC)-based immunotherapy, DCs are activated ex vivo and reinjected into the host to perform anti-cancer effector actions. DCs are antigen-presenting cells that process antigen material and present it on the cell surface to the T lymphocytes. After activation, T lymphocytes differentiate into different subtypes and migrate to destroy tumor cells or cells that have been infected by a virus^17–20^.

Mathematical models can be used to explain how cancer spreads, the immune system and the tumor interact, immune cells work, and to analyze the success of novel treatments and the combination of various therapies^21^. Examples of mathematical tools used to model living organisms are ordinary differential equations (ODEs) and agent-based models (ABMs)^22^. In ODE models, spatial and temporal dynamics cannot be simultaneously captured despite their simplicity. Additionally, these models do not consider stochasticity, although there are stochastic versions known as stochastic differential equations available. ABMs, as opposed to the conventional ODE modeling technique, provide more flexibility for individual action and are able to explain some of the behaviors observed in the tumor-immune system interaction, such as cell–cell interactions involved in the immune response, increased immune system access to tumor cells with tumor growth, and the impact of tumor antigen on the movement of immune cells within the TME^23^.

Many models in the form of ODEs and ABMs have been presented for tumor proliferation and describing the interaction between the tumor and the immune system along with various treatment methods. Carlos et al.^24^ modeled the interactions between immune system and tumor using ODE models, and used the model to test different DC vaccine injection protocols to find the amounts of DCs and injection times at which tumor treatment is significantly improved. Some studies such as^23,25^ show that DCs have nonlinear and random behavior and ABM models are more suitable than ODEs to model these behaviors. Macfarlane et al.^25^ proposed a discrete mathematical model of the interactions between immune system cells and monolayer tumor development. They investigated how cytotoxic T lymphocytes (CTLs) and DCs behave in the TME. The results indicated, the level of efficiency of T cells in destroying tumor cells is important and the immune response to cancer can be improved to some extent by raising the number of cytotoxic T cells, but an increase in the number of DCs may have the opposite effect. Pourhassanzadeh et al.^26^ introduced a novel two-dimensional random ABM to explore avascular tumor growth. In this model, the tumor is considered multilayer, and the behavior of the immune cells in the TME is investigated. Their results showed that immune cells are crucial in the prevention and treatment of cancer malignancy. Later, the authors offered a two-dimensional model of tumor growth with three layers^22^. Additionally, the concentration of oxygen, acid, and glucose in the TME was examined. The results showed that in the areas where there are tumor cells, oxygen is low and the TME becomes more acidic and leads to more aggressive tumor growth. However in this model, the immune system is not considered. Arabameri and Pourgholaminejad^23^ introduced a hybrid ABM-ODE model for studying adenosine’s role in immunotherapy. Their findings highlighted how crucial adenosine inhibition is to improve the immune system. However, in this model, the role of hypoxia and its impact on adenosine and tumor growth has not been incorporated.

To the best of the authors’ knowledge, no model has yet been proposed to quantify the relationship between hypoxia and adenosine, as well as the role of both in cancer immunotherapy. Therefore, the purpose of this study is to investigate the effect of oxygen deficiency on adenosine production and also the role of these two factors (hypoxia and adenosine) in providing tumor escape mechanisms in DC-based immunotherapy.

For the following reasons, we use a spatio-temporal model in this study: (1) the oxygen production or consumption in the TME is a function of location, that is, for example, a tumor cell consumes oxygen at a high rate, and immune system cells or normal body cells have a lower consumption rate, (2) an essential aspect of immune cell activity is how easily the immune system can access tumor cells, which depends on the geometrical pattern of the tumor’s outer layer. Therefore, in “Methods” section the proposed model is explained in detail. Then, in “Numerical simulations” section, numerical simulation of the model, finding the optimal vaccination pattern, and sensitivity identifiability analyses have been done. The findings are summarized and analyzed in “Discussion” section, along with a roadmap for future model improvements.

## Methods

**Figure 1:**
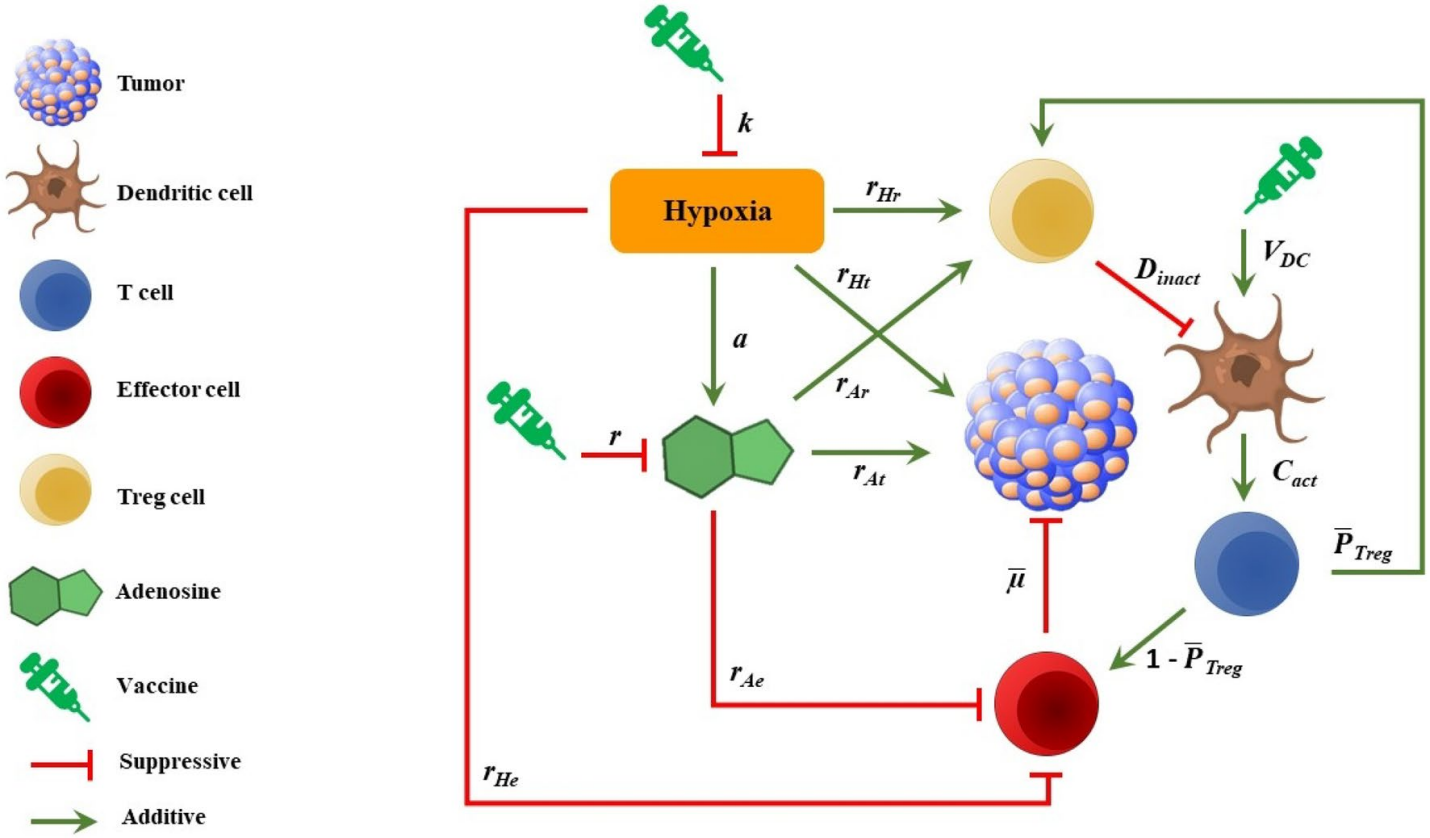
The suggested model’s overall structure. In this model, tumor, DC, T cell, Treg, effector cell, and factors such as hypoxia and adenosine are considered. The interaction of the factors with each other is shown by the arrows and their corresponding parameter is specified.

The overall structure of the model is depicted in Fig. 1. This diagram illustrates the interactions and connections between tumor, immune system cells, hypoxia, adenosine, and vaccines. While the model presented in this study has been calibrated using data specific to immunotherapy of breast cancer in mouse models, the interactions depicted in Fig. 1 are generally applicable to other types of solid tumors as well. We have made the assumption that there is a single population of inactive T cells, consisting of both naive Foxp3-expressing Tregs and naive CD8+ T cells. We have also assumed that this population can differentiate into both Tregs and effector cells. However, it is important to note that this is not the case in the immune system. This is done for the simplicity of the model and to reduce the number of variables. Hypoxia and adenosine increase the rate of tumor growth and decrease the cytotoxicity rate and the number of effector cells. Also, hypoxia has a positive effect on the production of adenosine. The vaccinations shown in this figure inhibit hypoxia and adenosine and increase the number of active DCs in TME.

### Modeling of tumor growth

We consider a two-dimensional discrete grid with size *N* × *N*. Time variable *t* is discretized with time step Δ*t* and the space variables *x* and *y* are discretized with space step Δ*x* = Δ*y* = *χ*. We employ a Moore neighborhood structure, in which every position in the lattice is surrounded by eight neighbors. Initially, we place a tumor cell in the center of this grid and it proliferates at rate *P*_*tumor*_. During division, the mother cell remains in its place and the daughter cell occupies one of the empty neighboring positions. If the tumor cell does not have an empty neighbor, it is not possible for it to divide^22,25^. As a result, only the cells comprising the outer layer of the tumor have the ability to proliferate, mirroring the conditions observed in the actual tumor environment. This reduction in effective growth rate has also been incorporated into previous studies^27^. It should be noted that we have omitted two phenomena related to the tumor for the sake of simplicity of our model: (1) the natural death of tumor cells, as the tumor experiences rapid growth in its early stages, making natural cell death negligible compared to its proliferation. (2) Metastasis, which typically does not manifest in the initial phases of tumor development. These assumptions have also been addressed in prior studies^23,25,28^.

### Modeling of immune cells

Immune cell mobility can now be more easily measured thanks to improvements in imaging techniques^25^. Before becoming activated, immune system cells are free to roam around in larger areas. These cells move across a limited area after activation. It has been demonstrated in Ref.^25^ that for a more precise representation of the movement of inactive immune cells, employing the Levy walk model is preferable, whereas the movement of active immune cells is more suitably characterized by Brownian motion. Therefore, in the vein of Ref.^25^, we consider Levy motion and Brownian motion for inactive and active immune system cells, respectively. The moving step in Levy walk is randomly generated from the cumulative distribution function:

1 $$F\left(z\right)={1-z}^{-l}, z=1, 2,\dots ,$$

where *z* is the number of movement steps in one direction and *l* is the walk exponent. After *z* is determined, a direction is randomly selected from empty neighbors. The cell will move in that time step and *z* − 1 time steps later in the selected direction. At each time step, the cell will not move if its selected path is occupied or out of the grid. Immune cells switch to Brownian motion when the tumor antigen activates them^25^. In this motion, at each time step there is an equal chance that the activated cells will move to one of the eight nearby grid sites. The cell can be moved when there is an empty space in its neighborhood and it is not outside the grid. The difference between these two motion types is the movement step. The cells in Levy walk take more steps in a certain direction^25^.

Four different types of immune cells are taken into account in our model: (I) DCs, (II) inactive T lymphocytes, (III) Treg cells, and (IV) effector cells. DCs and inactive T lymphocytes, whose numbers we denote by *N*_D_ and *N*_T_, respectively, are initially inactive and randomly distributed throughout the grid, moving in a Levy walk fashion. Please note that we have not considered the proliferation and death of immune cells in this model, nor have we included the healthy cell, similar to Ref.^25^. Moreover, cells are confined within the network and prevented from exiting. Upon nearing the network boundaries, they are repositioned in a direction opposite to the edge, employing either the Levy walk or the Brownian motion based on their movement prior to reaching the boundary.

**Figure 2:**
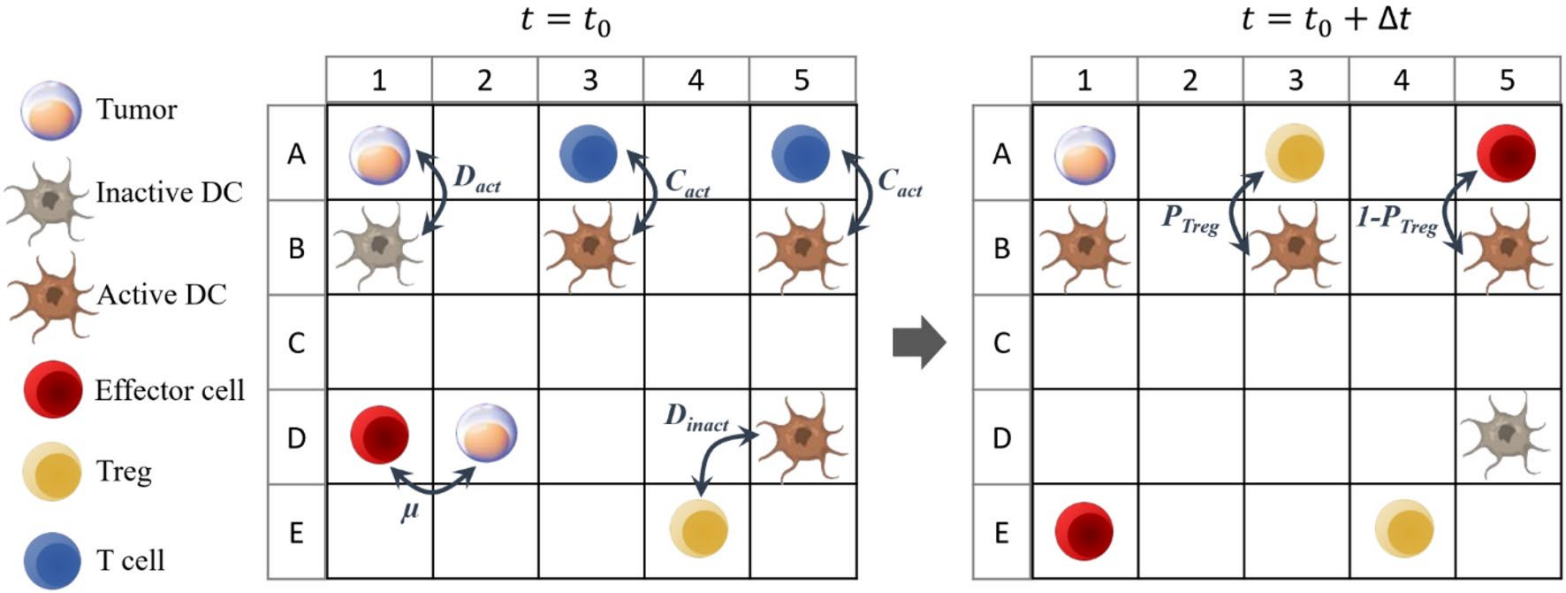
Interactions and communication between tumor and immune system cells in the discrete grid over time period Δ*t*. The tumor cell activates its neighboring inactive DC (**A1–B1**). The T cell next to the active DC becomes a Treg (**A3–B3**) or effector cell (**A5–B5**) after activation. The effector cell kills the tumor cell adjacent to it (**D1–D2**). The Treg cell inactivates its neighboring active DC (**D5–E4**).

Figure 2 displays a schematic example of a cross-sectional portion of the ABM before and after a time step Δ*t*. Inactive DCs may become active in interaction with cancer cells. If an inactive DC is next to a cancer cell (Fig. 2A1–B1), it will be activated at rate *D*_act_ and then it moves in the grid with the Brownian motion. An activated DC can activate the inactive T lymphocytes located in its neighborhood at rate *C*_act_ (Fig. 2A3–B3,A5–B5). It should be noted that immunogenicity or tolerogenicity of DCs depends on the environmental conditions during their maturation process^29^. During activation, T-lymphocytes convert to Treg at rate *P*_Treg_ (Fig. 2A3–B3) or effector cells at rate 1−*P*_Treg_ (Fig. 2A5–B5). An activated DC can activate several T lymphocytes. The parameter *P*_Treg_ can be considered as the tolerogenicity of activated DCs. Effector cells destroy their neighboring tumor cells at rate *μ* (Fig. 2D1,D2). At every time step, each effector cell has the ability to eliminate a tumor cell situated in its neighborhood. If there is more than one tumor cell in the neighborhood of the effective cell, one of them is randomly selected and removed with probability *µ*. Subsequently, when a tumor cell is eliminated, the corresponding location is designated as an empty space. It is shown that effector cells can make multiple contacts and kill serially in a time-dependent manner^30^. Therefore, we assume that the effector cell can continue to move and kill cancer cells again. Active DCs located in the neighborhood of a Treg are deactivated at rate *D*_*inact*_ (Fig. 2D5–E4).

### Hypoxia and adenosine modeling

To model the relationship between oxygen and hypoxia, it is apparent that a decrease in oxygen concentration leads to a reduction in enzymatic reactions. Based on the relationships depicted in Fig. 5 of Ref.^31^, one can deduce a straightforward relationship: the hypoxia level is inversely proportional to the oxygen concentration. Therefore, in all grid sites, we considered hypoxia to be the inverse of oxygen concentration. We use a discretized diffusion equation to update the oxygen concentration at all sites of the grid in each time step. To reduce the required computation, we used the method presented in Ref.^22^. In each time step, the grid is divided into 3 × 3 non-overlapping blocks and the concentration of oxygen at the center of each block is calculated as below:

2 $${U}_{m,n}^{t+1}={\alpha }{\times A}_{m,n}^{t}+\left(1-\alpha \right)\times {U}_{m,n}^{t}-{f}\left(n,m,t\right)+ kpx,$$

where

3 $${A}_{m,n}^{t}=(\sum_{j=-1}^{j=1} \sum_{k=-1}^{k=1}{U}_{m-j, n-k}^{t})/9,$$

and ${U}_{mn}^{t}$ represents the concentration of oxygen and* m*, *n*, and *t* denote the column number of the spatial position, row number of the spatial position, and the time step, respectively. ${f}(n,m,t)$ is a consumer or producer function and $f\left(m,n,t\right)=\overline{f}I\left(m,n,t\right)$, where $\overline{f}$ is a constant and $I\left(m,n,t\right)$ is an indicator function with value 1 in tumor sites and 0 elsewhere. The parameter *α* ϵ (0,1) represents the diffusion coefficient and parameter *k* is related to the effect of the vaccine on oxygen. Hypoxia inhibitor vaccine is modeled in (5). After calculating the concentration of oxygen at the center of each 3 × 3 block, the calculated value is replaced and redistributed in the whole block.

In the next step, adenosine and the effect of hypoxia on adenosine are modeled as follows:

4 $$\frac{dAd}{dt}= \beta +\frac{a}{U+c}-rAd-\gamma sAd.$$

The level of adenosine production in cells can vary depending on the specific cell type and physiological conditions. Generally, there is a base level, denoted as *β*, for adenosine production rate^32^. However, under certain circumstances such as low oxygen levels, the production of adenosine can exceed this base level. This increase in adenosine production serves as a protective response to the decreased oxygen availability^33^. The parameter $a$ adjusts the strength of adenosine production by hypoxia. As mentioned before, we have considered that the hypoxia level is inversely proportional to the oxygen concentration. We have added a near-zero value (the parameter *c*) to the denominator to avoid division by zero. The parameter *r* is the elimination rate of adenosine, and $\gamma$ models the adenosine inhibition by vaccine. The parameters *β*, *a*, and *r* are fine-tuned to closely match the experimental data on adenosine dynamics, as illustrated in Fig. 5 of Ref.^14^. The parameter *γ* is estimated using experimental data to guarantee that the levels of adenosine show proportional changes both before and after inhibitor administration, closely matching empirical data of Ref.^3^. Adenosine inhibitor vaccine is modeled in (6). This vaccine reduces the level of adenosine. We will now consider two simple ODEs that represent the concentration of adenosine and hypoxia inhibitor vaccines:

5 $$\frac{dpx}{dt}=v\left(t\right)- \eta px,$$

6 $$\frac{ds}{dt}= {v}_{s}\left(t\right)-\lambda s.$$

In (5), *px* represents the normalized concentration of hypoxia inhibitor (unitless), and *η* is the drug elimination rate. The parameter *η* is estimated to match experimental data by considering pre- and post-vaccine hypoxia levels^4^. In (6), *s* represents the normalized concentration of adenosine inhibitor (unitless), and *λ* is its elimination rate. The parameters of the adenosine inhibitor drug are estimated based on experimental data, where the drug levels were measured at different time points following injection. For further information, please refer to Fig. 1 in Ref.^3^. $v\left(t\right)=40\delta (t-{t}_{v})$ and ${v}_{s}\left(t\right)=4200\delta (t-{t}_{vs})$ are the vaccination functions for hypoxia and adenosine inhibitor drugs respectively. *t*_*v*_ and *t*_*vs*_ are the vaccination time points for hypoxia and adenosine inhibitor drugs respectively. For the sake of simplicity, we have omitted the inclusion of relationships to simulate the diffusion of these drugs within the tumor environment, assuming a uniform concentration across all grid cells at each time point.

Adenosine inhibitor drug has adhesive properties and accumulates in the tumor area^3^. Therefore, a moving average filter is considered to model that properly by:

7 $${S}_{t}\left[n\right]=\frac{1}{M}{\sum }_{k=0}^{M-1}s[n-k].$$

Equation (7) means that a moving average filter is applied to the output of (6). In (7), *M* is the number of points used for averaging (see the output of (5) and (7) in Fig. A2 in Appendices).

Adenosine and hypoxia have been shown to act as pro-tumor factors through various mechanisms. One of these mechanisms is the promotion of Treg activation^3,4^. Therefore, we will consider the following simple ODE to model the probability of Treg activation rate:

8 $${\overline{P} }_{Treg}={P}_{Treg}+\frac{{r}_{Hr}}{U+c}{+ r}_{Ar} Ad,$$

where the production rate of Treg cells $({\overline{P} }_{Treg}$) increases with decreasing oxygen concentration or rising adenosine at rates *r*_*Hr*_ and *r*_*Ar*_, respectively. ${P}_{Treg}$ is the base value of Treg activation rate.

Secondly, adenosine and hypoxia have been shown to directly promote the proliferation rate of tumor cells^3,4,7,9^:

9 $${\overline{P} }_{Tumor}={P}_{Tumor}+\frac{{r}_{Ht}}{U+c}{+ r}_{At} Ad,$$

where* r*_*At*_ and *r*_*Ht*_ are related to the effect of adenosine and hypoxia on the production rate of tumor. ${P}_{Tumor}$ is the base value of tumor proliferation rate.

Thirdly, adenosine and hypoxia have been found to decrease the cytotoxicity rate of effector cells^3,4^:

10 $$\overline{\mu }=\mu - \frac{{r}_{He}}{U+c}{- r}_{Ae} Ad.$$

Similarly, in (10) *r*_*Ae*_ and *r*_*He*_ are related to the effect of adenosine and hypoxia on the effector cells cytotoxicity rate. *µ* is the base value of effector cell cytotoxicity.

The last step is DC modeling. *V*_*DC*_ number of active DCs was randomly distributed in the grid on the seventh day (the injection day). This number was selected such that the model output corresponds to the experimental results.

## Numerical simulations

### Set-up of numerical simulations

We consider a 210 × 210 grid for our model. The size of the grid is chosen so that there is enough space for the tumor to grow until the 18th day (the last day of experimental data). The number of DCs and lymphocytes in the body has been extensively studied in various references. However, it is important to note that these ratios can be altered when cancer is present, and the variations can depend on the specific characteristics of the immune system^34^. Therefore, when selecting the cell counts for this model, we consider the available data set and the desired output of the model. As a result, on the first day, we randomly distribute 100 inactive DCs and 750 inactive T lymphocytes across the entire grid.

The experimental data used in this study includes the percentage of Treg cells in various groups with and without treatment. In order to convert the unit of this data into the number of Treg cells, a coefficient was applied. This coefficient was calculated to ensure that the variable representing Treg cell count matched between the simulation and experimental data for the untreated group. The total experimental data pertaining to Treg cells was then multiplied by this coefficient.

The average diameter of various tumor cells is reported in Refs.^35–37^. Based on these studies and to reduce complexity in the simulations, we assume that each grid-site is 10 µm apart in both the *x* and *y* directions. Therefore, the size of each grid-site is 10^–4^ mm^2^. To determine the tumor volume, the number of sites occupied by the tumor is counted and then multiplied by 10^–4^ mm^2^. To convert the unit of tumor size from the number of cells to mm^3^, we multiply it by 2200. We selected this factor based on the observation that significant volumetric expansion occurs in three dimensions compared to two dimensions and one dimension, as demonstrated in Ref.^38^. This study measured and compared tumor volume when cells were arranged in one dimension (on a line), two dimensions (in a plane), or in a three-dimensional space, and a significant increase in volume in three dimensions, compared to two-dimensional and one-dimensional found. Therefore, we chose this factor so that the model and real outputs in the first step of the simulation in the untreated group match. We chose a time step of Δ*t* = 2 min to simulate our model (small enough to capture the dynamics of immune cell movements).

We hold the boundaries of the grid at a fixed oxygen concentration (0.8 mMol) and consider a zero initial condition for the concentration of oxygen inside the grid. It is assumed that there are blood vessels on the borders and they diffuse oxygen into the grid (see the simulation steps of the model in Fig. A4 in Appendices). The range of some model parameters has been determined based on previous studies. By utilizing experimental data, these parameters have been adjusted to ensure that the outputs of the model closely match the experimental data. The estimated parameters are listed in Table 1. For a visual representation of a sample simulation in the lattice of the proposed model, please refer to Fig. A1 in Appendices. The code pertaining to siRNA + Px-478 + DC group is available on GitHub at: https://github.com/elaheghiyabi96/Modeling-Tumor-Escape-Mechanisms-for-DC-Based-Immunotherapy-Insights-into-the-Role-of-Hypoxia-and-A.

**Table 1:** The parameters in the model, their description and their values estimated from experimental data.

	Parameter	Description	Value	Unit	Source
1	$\gamma$	Effect of the vaccine on adenosine	0.000038	Δ*t*^−1^*	Estim
2	$a$	Effect of hypoxia on adenosine	0.9412	Mol. cells^−1^	–
3	$r$	Adenosine elimination rate	0.07	Δ*t*^−1^	Estim
4	$\beta$	Constant rate of adenosine production	12	Δ*t*^−1^	Estim
5	*r*_*At*_	Effect of adenosine on tumor	0.000025	Δ*t*^−1^	–
6	*r*_*Ht*_	Effect of hypoxia on tumor	0.0021	Mol. cells^−1^. Δ*t*^−2^	Estim
7	*r*_*Ar*_	Adenosine effect on Treg	0.0015	Cells. Δ*t*^−1^	Estim
8	*r*_*Hr*_	Hypoxia effect on Treg	0.0047	Mol. cells^−2^. Δ*t*^−2^	Estim
9	$\lambda$	The elimination rate of adenosine inhibitor vaccine	0.002	Δ*t*^−1^	Estim
10	*D*_*act*_	DC activation rate	0.003	Cells. Δ*t*^−1^	From^25^ with change
11	*C*_*act*_	T lymphocyte activation rate	0.24	Cells. Δ*t*^−1^	From^25^ with change
12	*µ*	Effector cell cytotoxicity rate	0.45	Cells. Δ*t*^−1^	From^25^ with change
13	*r*_*Ae*_	Effect of adenosine on effector cell cytotoxicity rate	0.001	Δ*t*^−1^	Fit to data
14	*r*_*He*_	Effect of hypoxia on effector cell cytotoxicity rate	0.014	Mol. cells^−2^. Δ*t*^−2^	Fit to data
15	*D*_*inact*_	DCs inactivation rate by Treg	0.01	Cells. Δ*t*^−1^	Fit to data
16	$\eta$	Elimination rate of hypoxia inhibitory vaccine	0.014	Δ*t*^−1^	Estim
17	*K*	The effect of the vaccine on oxygen	0.00004	Mol. cells^−1^. Δ*t*^−1^	Estim
18	*P*_*Treg*_	Treg cell production rate	0.3	Cells. Δ*t*^−1^	Fit to data
19	*P*_*tumor*_	Tumor growth rate	0.001	Δ*t*^−1^	^25^
20	${\overline{f}}$	Rate of oxygen consumption by the tumor cell	0.0023	Mol. cells^−1^. Δ*t*^−1^	From^22^ with change
21	*c*	To avoid division by zero in the denominator	0.01	Mol. cells^−1^. Δ*t*^−1^	–
22	*L*	Walk exponent	1.15	–	^25^
23	*N*_*T*_	T-lymphocyte count	750	–	–
24	*N*_*D*_	Inactive DC count	100	–	–
25	*V*_*DC*_	DC vaccine	70	–	–
26	*M*	number of points in moving average	800	–	–
27	*N*	size of grid points in both the x and y directions	210	–	–
28	*Χ*	size of each grid-site in both the x and y directions	10	μm	^25^

*Δ*t* = 2 min.

### Model assessment

We investigate the dynamics of different variables in the model in 8 groups (see Table 2). Adenosine and hypoxia inhibition have been investigated in two different studies using siRNA-loaded chitosan lactate nanoparticles (siRNA for short) and Px-478 vaccines^3,4^. In both studies, tumors were established by injection of 7 × 10^5^ 4T1 breast cancer cells subcutaneously into the right flank of female BALB/c mice (6–8 weeks old) on day 0, and tumor size (mm^3^) was assessed every 2 days after that. Also, both works used DC-based immunotherapy in combination with inhibition of adenosine or hypoxia. The DC vaccine was injected into mice on day 7, and the tumor size is available up to day 18 in both works. In addition to tumor size, some other variables such as the number of Treg cells, cytotoxicity of effector cells, or the concentration of vaccines in tumor area were also measured on specific days.

**Table 2:** Different groups in experimental and computational investigations of this work.

	Group name	Px-478 (Days = 6, 8, 10, 13, 15)	siRNA (Days = 6, 9, 12, 15)	DC (Day = 7)
1	Untreated	×	×	×
2	Px-478	✓	×	×
3	siRNA	×	✓	×
4	DC	×	×	✓
5	Px-478 + DC	✓	×	✓
6	siRNA + DC	×	✓	✓
7	siRNA + Px-478	✓	✓	×
8	siRNA + Px-478 + DC	✓	✓	✓

Since our model is stochastic, we repeat each simulation 30 times and store the results, and then report the average and 95% confidence interval of stored results. For a quantitative comparison, we report R-squared (RSQ) for each group. The RSQ is calculated to assess how well the model output and the empirical data match and it has a value between 0 and 1 (the larger it is, the closer the model output and the empirical data are). In this study, our primary objective is to investigate tumor behavior during the initial days following tumor establishment, with a specific emphasis on analyzing the impact of vaccines using experimental data. Given that extending simulations may not yield additional insights relevant to our research question, our simulations are confined to the same timeframe as the experimental data collection (up to 18 days post tumor establishment).

**Figure 3:**
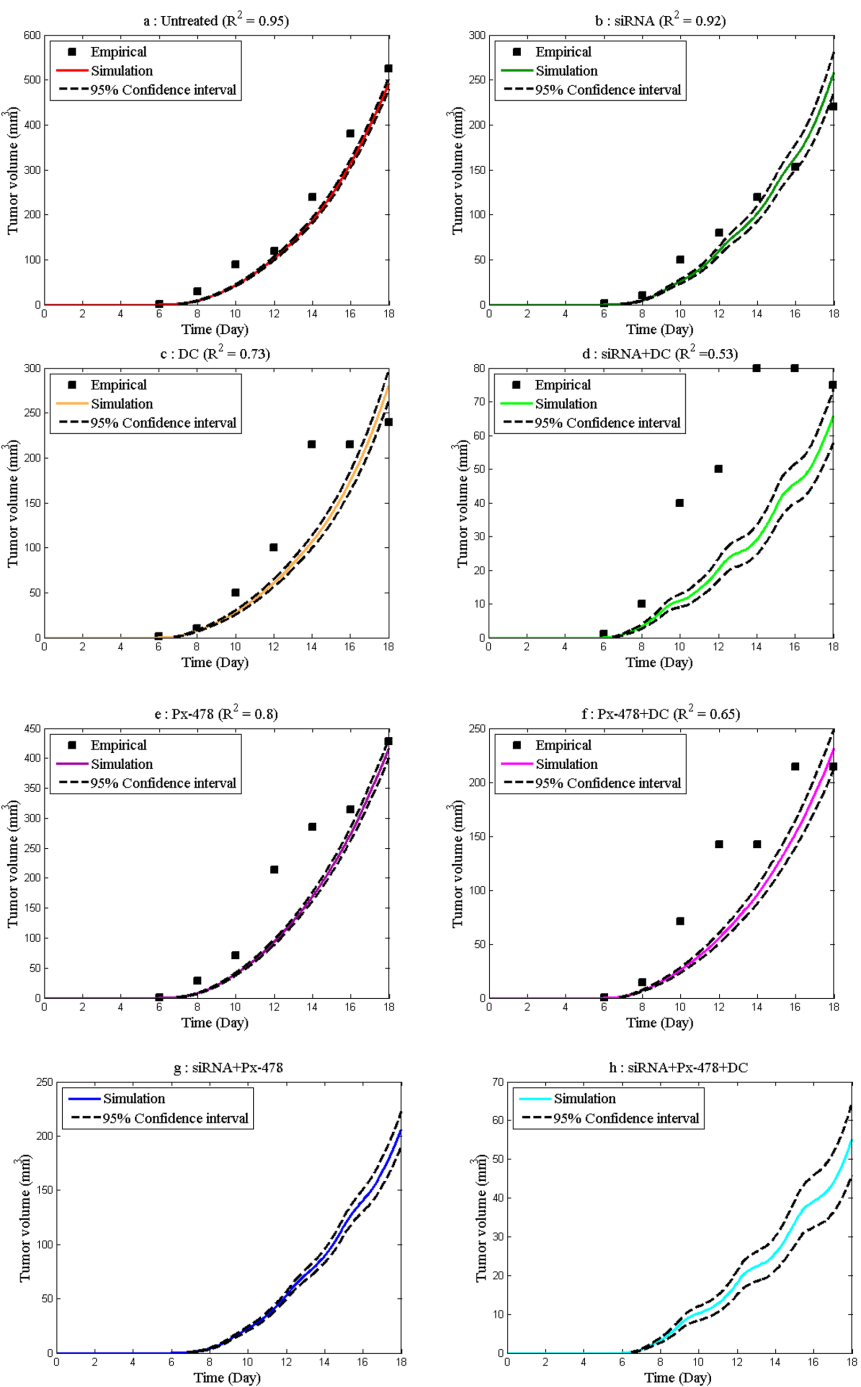
Tumor volume was predicted by the model and measured in the empirical data^3,4^ in different groups. (**a**) Untreated. (**b**) Treatment with adenosine inhibitor. (**c**) Treatment with DC vaccine. (**d**) Treatment with adenosine inhibitor and DC vaccine. (**e**) Treatment with hypoxia inhibitor. (**f**) Treatment with hypoxia inhibitor and DC vaccine. (**g**) Treatment with adenosine and hypoxia inhibitor. (**h**) Treatment with adenosine and hypoxia inhibitor and DC vaccine.

Figure 3 shows the volume of the tumor in different groups. In the plots on this figure, the squares show the empirical data and the dashed lines show the confidence interval. According to these plots, the groups that had combined treatment are more effective than the groups that had single treatment, and the most effective treatment is the combined treatment with all vaccines. There was no experimental data for the group’s siRNA + Px-478 and siRNA + Px-478 + DC, and conclusions were made based on the suggested model. The best RSQ corresponds to untreated group and the worst RSQ corresponds to treatment with siRNA + DC group. Although the value of RSQ has decreased in some groups, but by comparing the behavior of the model in different groups, it can be concluded the model has correctly predicted the improvement or deterioration of the results in different groups. For a more detailed comparison and analysis of the results obtained from the model and experimental data in different groups, please see Fig. A3 in the Appendixes.

**Figure 4:**
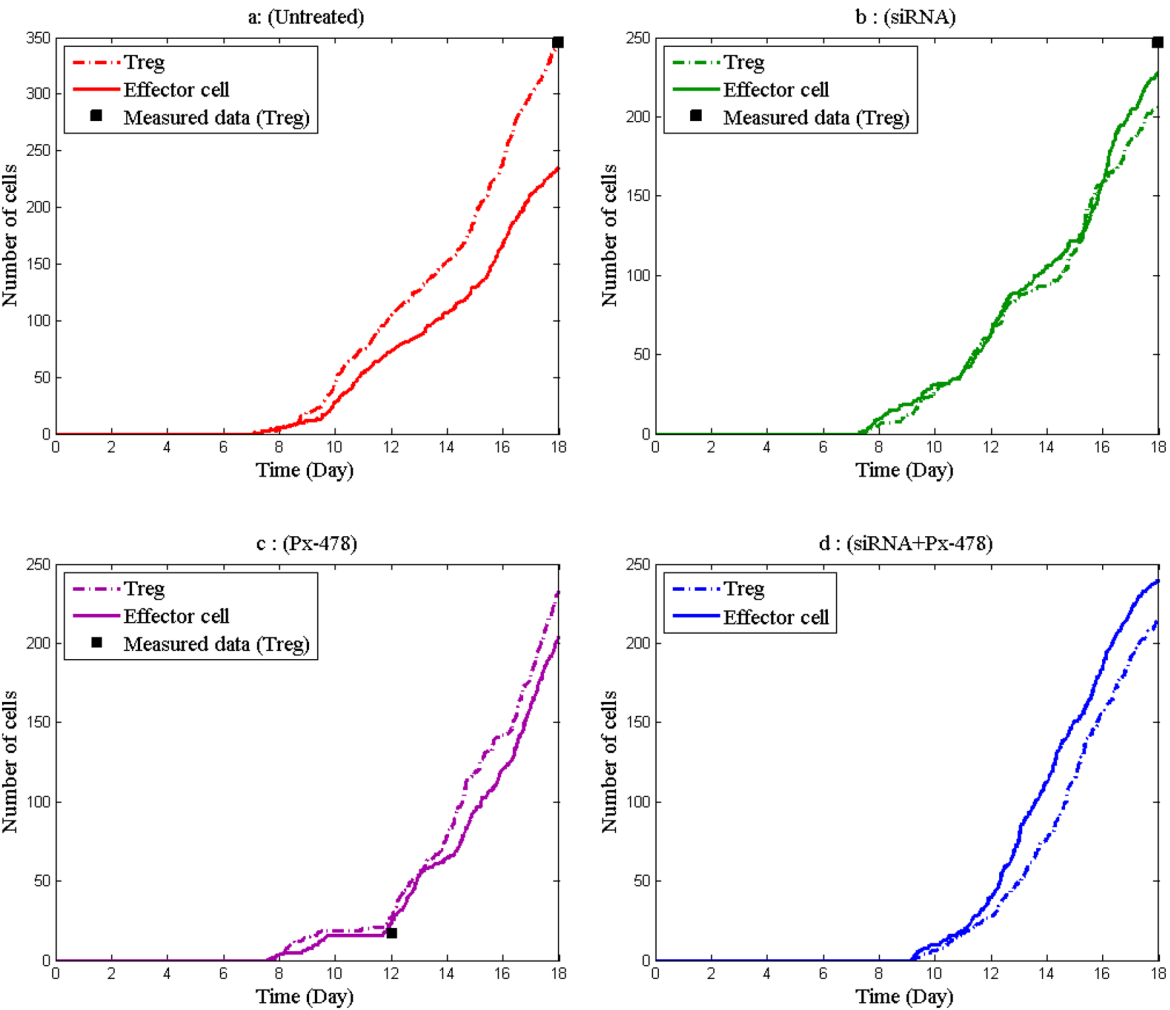
The number of effector cells and Treg cells in different groups. (**a**) Untreated. (**b**) Treatment with adenosine inhibitor. (**c**) Treatment with hypoxia inhibitor. (**d**) Treatment with adenosine inhibitor and hypoxia inhibitor.

Treg/effector cell ratio correlates with protective immunity in mice and humans^39^. To investigate this ratio, Fig. 4 shows the number of effector cells and Tregs in four groups. As we wanted to see the effect of only adenosine and hypoxia on effector and Treg cell numbers, the groups that received the DC vaccine were not considered here. According to Fig. 6, in the siRNA and Px-478 groups (Fig. 4b,c), the difference in the number of Treg and effector cells has decreased compared to the untreated group. Because adenosine and hypoxia have been suppressed, immune suppressor cells have reduced in siRNA and Px-478 groups. However, when the outcomes are compared, we can find that the adenosine inhibitor has enhanced the Treg/effector cell ratio more than the hypoxia inhibitor. In the Px-478 + siRNA group, the number of effector cells has increased more than the number of Treg cells (Fig. 4d). Therefore, the outputs demonstrate that combination therapy is better than other groups in improving immune system cell performance.

**Figure 5:**
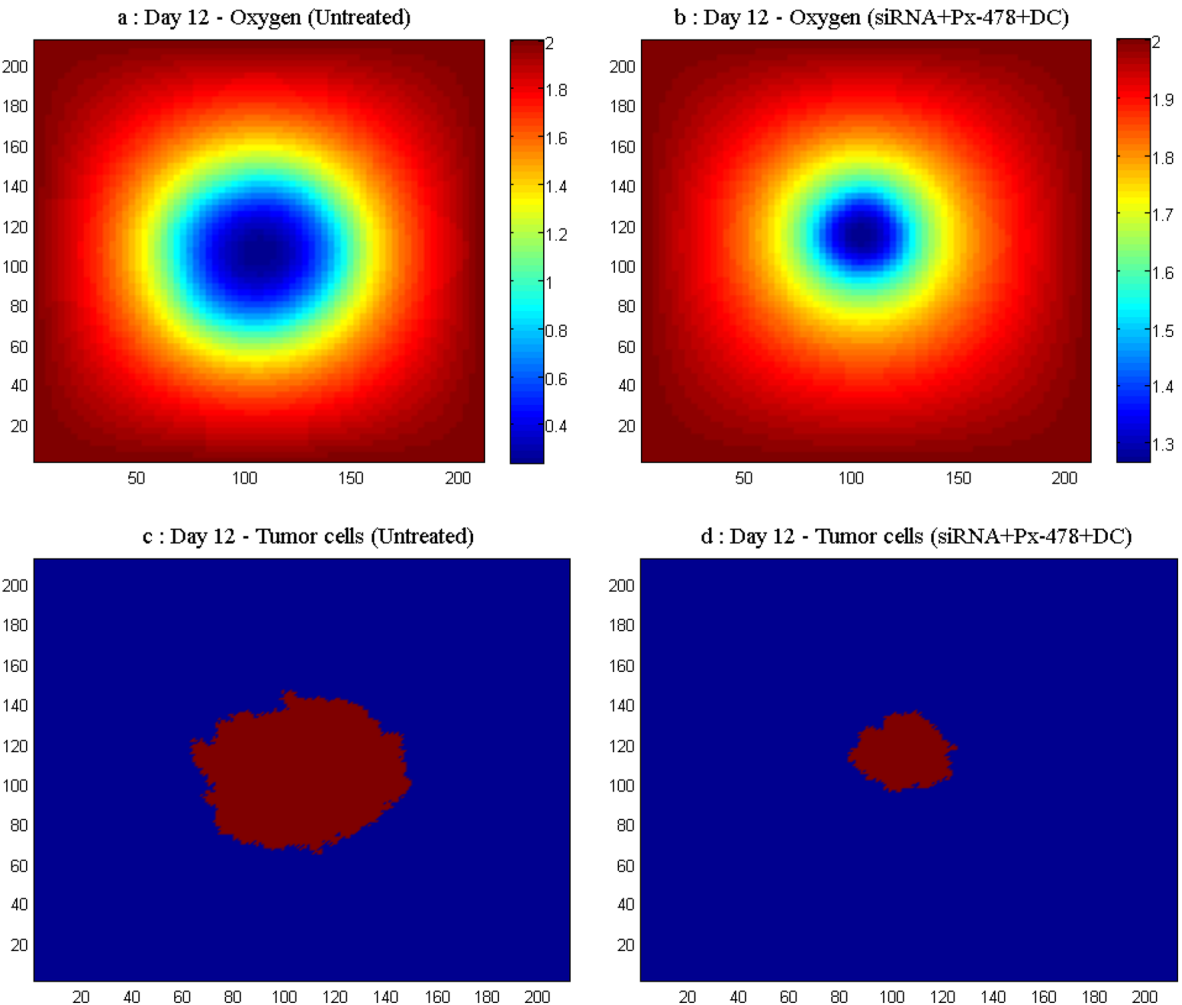
Comparison of the oxygen concentration and tumor cells in the untreated and siRNA + Px-478 + DC groups on day 12. (**a**) Oxygen concentration in the untreated group on day 12. (**b**) Oxygen concentration in the siRNA + Px-478 + DC group on day 12. (**c**) Tumor cells in the untreated group on day 12. (**d**) Tumor cells in the siRNA + Px-478 + DC group on day 12.

Figure 5 compares the oxygen concentration and tumor cells distribution in the untreated and the siRNA + Px-478 + DC groups on day 12. The oxygen concentration in siRNA + Px-478 + DC group has increased compared to the untreated group in the surrounding areas of the tumor, and as a result, hypoxia decreased. Also, the tumor volume has decreased in the siRNA + Px-478 + DC group.

**Figure 6:**
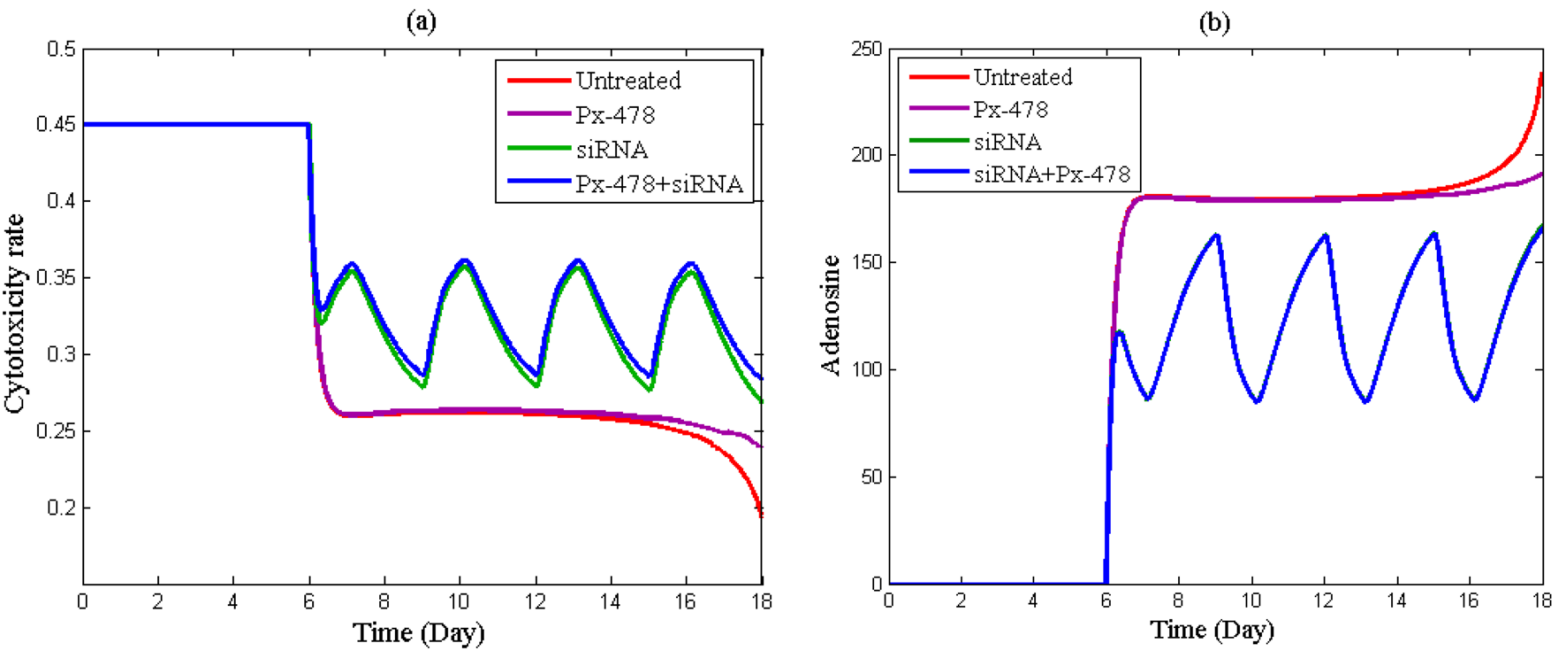
Investigating the time course of effector cell cytotoxicity rate and adenosine concentration in four groups. (**a**) Effector cell cytotoxicity rate, (**b**) the adenosine concentration.

Figure 6a shows the dynamic of *µ* (effector cell cytotoxicity rate) in different treatment groups. The fluctuation of the output in siRNA and siRNA + Px-478 groups of this Figure is due to the periodic injection of the vaccine. As is seen in this figure, in the untreated group, tumor development and increasing hypoxia and adenosine have decreased cytotoxicity rate over time. In the Px-478 and siRNA groups, the cytotoxicity rate has increased. Hypoxia and adenosine reduce the effector cell cytotoxicity rate. Therefore, their inhibiting has prevented the reduction of this rate. In comparison to others groups, the cytotoxicity rate in the Px-478 + siRNA group (combination therapy) is higher. Therefore, inhibition of hypoxia and adenosine has improved the function of effector cells.

Figure 6b shows the level of adenosine in different groups. Adenosine has begun to rise more quickly in the untreated group after the 14th day, because hypoxia in the TME increases as the tumor grows. Adenosine concentration in the TME also rises when hypoxia increases. In the Px-478 group, suppression of hypoxia prevents the acute increase in adenosine. In Fig. 6b the results of the siRNA and siRNA + Px-478 groups are identical. Because according to the outputs of tumor volume and immune system cells, adenosine has a greater impact on tumor growth. As a result, the tumor volume is reduced when adenosine is inhibited. As tumor volume decreases, hypoxia also decreases and a low amount of hypoxia cannot have a substantial effect on the growth of adenosine.

### The effect of changing the injection dose and time

**Figure 7:**
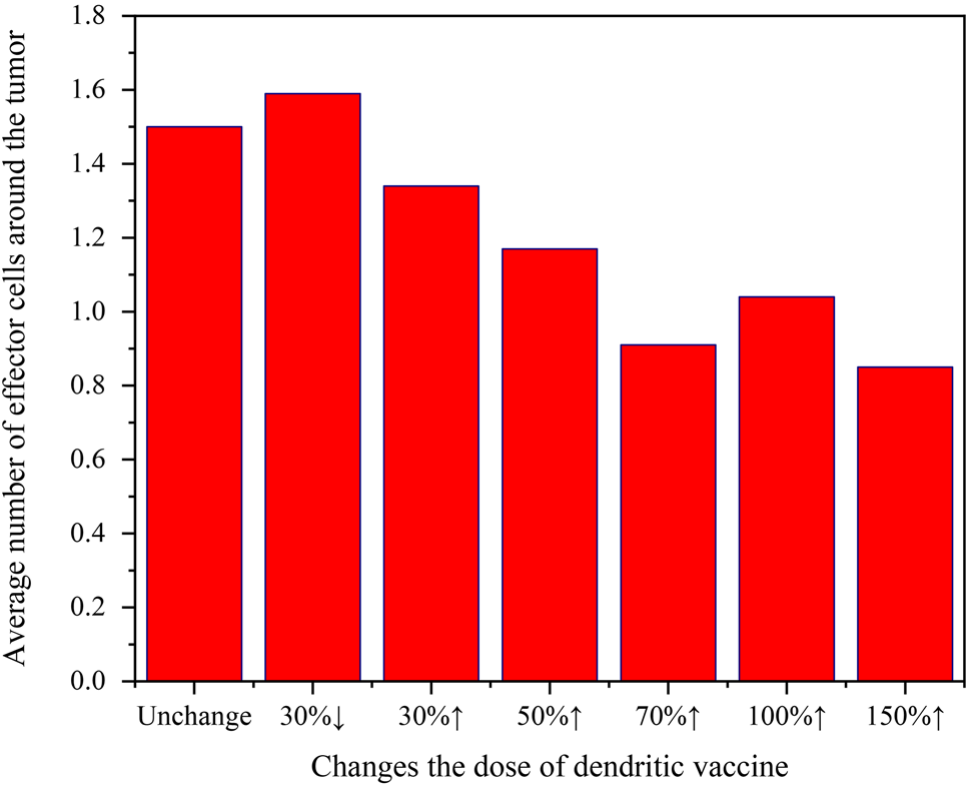
The average number of effector cells around the tumor as a result of changing the dose of the DC vaccine.

The proposed model may be used to monitor changes in tumor volume by varying the time, number, and dose of vaccines. We have observed that the siRNA + Px-478 + DC group yields the most favorable therapeutic outcomes (see Fig. 3h). The results of altering the injection patterns within this group are presented in Table 3. In the first type of these patterns, only the dose and number of injections of the DC vaccine were altered, and all other vaccinations remained unchanged compared to the siRNA + Px-478 + DC group. The results of this type emphasize how crucial it is to choose the correct dose and number of injections. It can be seen that the sooner the vaccine is injected, the more effective it is and the less the tumor grows. An interesting result of this type is that increasing the dose and number of injections may sometimes unexpectedly lead to an increase in tumor size. Because the increase of active DCs may cause traffic in the area around the tumor, preventing effector cells from reaching the tumor cells. To investigate this effect, the average number of effector cells surrounding the tumor was measured for 18 days, and the results are displayed as a function of injections dose in Fig. 7. As seen in this figure, increasing the number of injected cells decreases the access of effective cells to tumor cells. This is also reported in other experimental^40^ and computational^25^ studies.

**Table 3:** Investigating the effect of changes in the dose, the time of the first injection, and the time interval between injections on the final and the average volume of the tumor.

Type of vaccine	Type of change		Average tumor volume (mm^3^)	Tumor volume on day 18 (mm^3^)
(1) DC vaccine	Time of initial injection (one injection only)	★Day 6	0.0005	0
		Day 7	21.5	64.25
		Day 8	35.23	102.69
	Injection dose (injection on day 7)	30%↓	25.4	74.2
		30%↑	16.9	41.8
		50%↑	21.1	64.35
	The number of injections (injection starts on day 7 and other injections with an interval of one day)	2 times	15.16	43.16
		4 times	26.9	68.9
		6 times	33.97	85.77
	The number of injections (injection starts on day 7 and other injections with an interval of two day)	2 times	29.45	82.47
		4 times	28.37	80.6
		6 times	25.04	66.28
(2) siRNA vaccine	Changing the days of siRNA vaccine injection (other vaccines are unchanged)	Everyday	9.89	16.47
		Every two days	19.93	44.55
		Every three days	22.22	59.1
	Changing the dose of siRNA vaccine (other vaccines are unchanged)	30%↓	41.19	121.83
		30%↑	24.43	55.63
		50%↑	16.96	39.68
(3) Px-478 vaccine	Changing the days of Px-478 vaccine injection (other vaccines are unchanged)	Everyday	16.7	41.8
		Every two days	20.1	61.20
		Every three days	31.57	69.41
	Changing the dose of Px-478 vaccine (other vaccines are unchanged)	30%↓	27.36	77.24
		30%↑	24.6	58.9
		50%↑	21.28	58.87
(4) Px-478 and siRNA vaccine	Changing the dose of Px-478 and siRNA vaccines and the time of injection of these vaccines every day	30%↑	3.02	0
		50%↑	2.6	0
		70%↑	1.2	0
	Changing the dose of Px-478 and siRNA vaccines and the time of injection of these vaccines every two days	30%↑	14.95	34.56
		★50%↑	2.6	4.6
		70%↑	2.9	3.36
	Changing the dose of Px-478 and siRNA vaccines and the time of injection of these vaccines every three days	30%↑	16.48	47.96
		50%↑	13.58	31.13
		70%↑	9.9	21.53
	Injection of Px-478 and siRNA vaccines every other day	Start with Px-478	21.42	48.37
		★Start with siRNA	8.6	18.43

In the second to forth part of Table 3, different patterns for the injection of hypoxia and adenosine-inhibiting vaccines have been simulated. The outcomes demonstrate that increasing the injection volume and decreasing the time between injections can lead to improved treatment. But this improvement may be insignificant in higher doses or higher number of injections. When the vaccines are given every other day, the result is more favorable when the adenosine vaccine is injected first. Also, the results show that adenosine has a greater effect on tumor growth than hypoxia. According to the results of Table 3, only a few of the tested scenarios significantly reduced the tumor volume and are more effective than the others: (1) injection of DC vaccine on day 6, (2) injection of siRNA and Px-478 vaccines once and every two days with a 50% dose increase, (3) injection of Px-478 and siRNA vaccines every other day when the siRNA vaccine is injected first.

### Global sensitivity analysis

**Figure 8:**
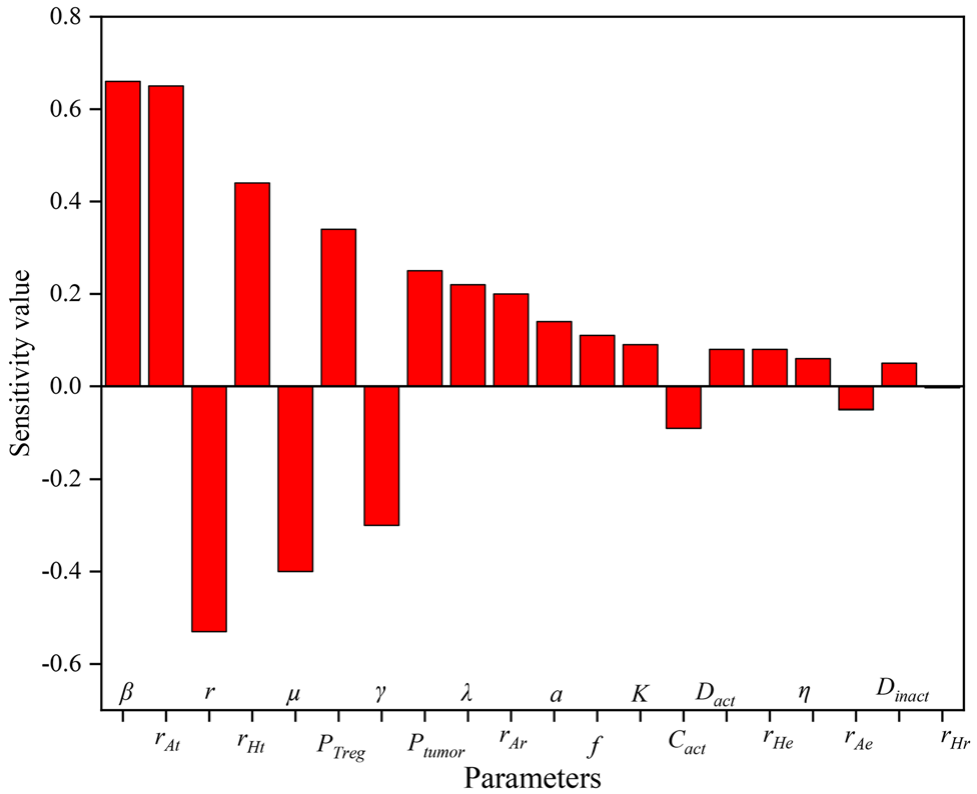
Sensitivity value of the model parameters.

Global sensitivity analysis calculates how sensitive the model outputs are to the model parameters. Finding the model-sensitive parameters enables us to identify the crucial and influencing agents in TME. Then, using these important factors, the model and treatment techniques may be improved and expanded. Unlike local sensitivity analysis, global sensitivity analysis involves changing every model parameter at once. Since all input parameters can be changed at once, the global sensitivity analysis is more realistic and ideal for nonlinear input–output relationships. The sensitivity analysis results are shown in Fig. 8. The output considered in this analysis is the average tumor volume during 18 days (please refer to the sensitivity analysis method provided in the Appendices). According to this figure, the most sensitive parameters are *β*, *r*_*At*_, and *r*. These parameters are related to the constant rate of adenosine production, the effect of adenosine on tumor growth and, the rate of adenosine elimination, respectively. This emphasizes that in this model, adenosine has a greater impact on output than hypoxia. Also, in the preceding section, the effects of the adenosine inhibitor vaccine on tumor volume were more visible than hypoxia.

**Figure 9:**
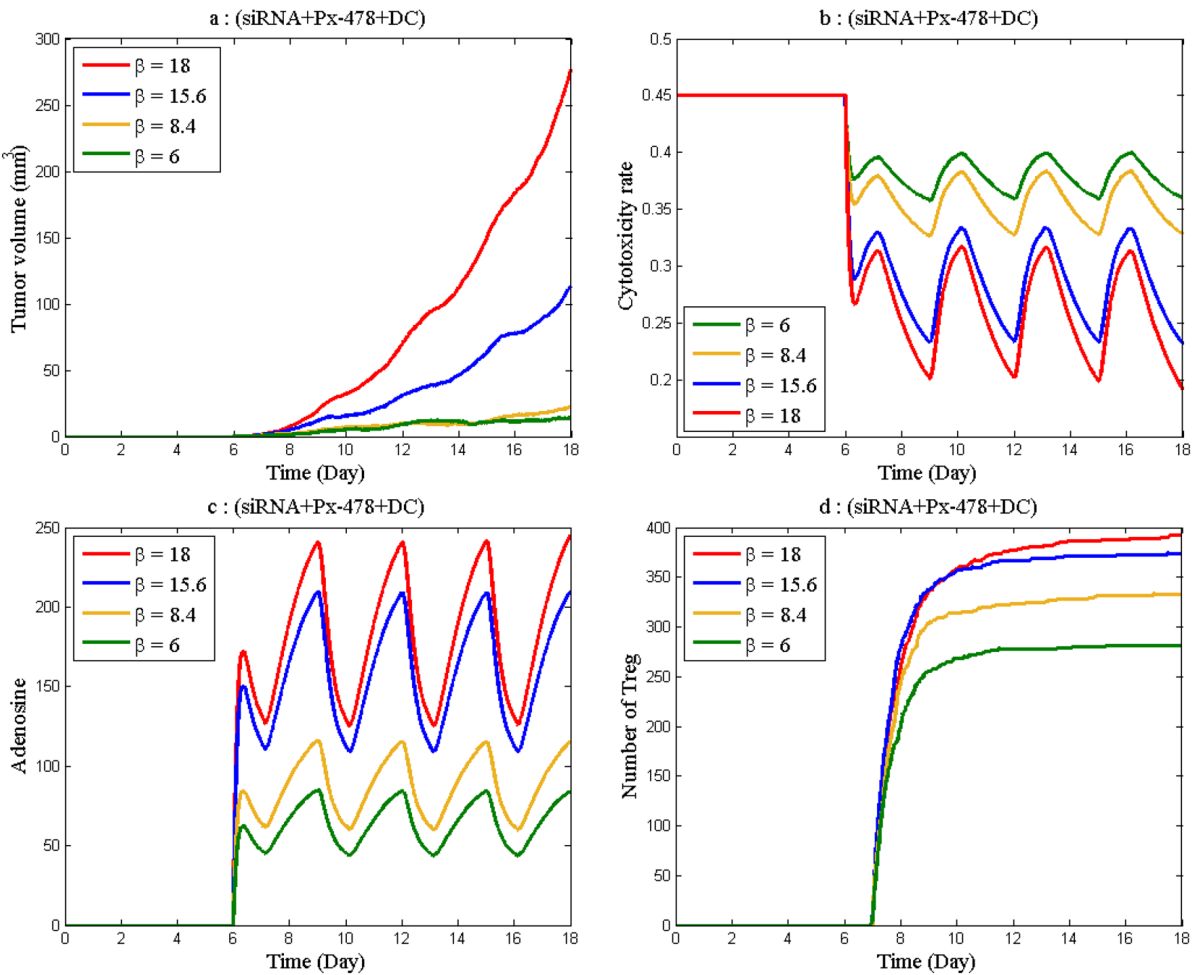
The time course of tumor volume, effector cell cytotoxicity rate, adenosine concentration, and number of Treg as a result of changes in *β* (**a**) Tumor volume. (**b**) Effector cell cytotoxicity rate. (**c**) Level of adenosine. (**d**) Number of Treg*.*

According to the sensitivity analysis, *β* (constant rate of adenosine production) is the most sensitive parameter of the model. To investigate how this parameter affects the variables of the model, the time evolution of the tumor, the killing rate of the effective cells, adenosine, and number of Treg for four values of *β* are plotted in Fig. 9. By just raising the constant rate of adenosine production, the level of adenosine, tumor volume, and number of Treg are significantly enhanced while the cytotoxicity rate is reduced. This demonstrates that adenosine plays an important role in tumor growth and immune system activity. Inhibiting adenosine or other factors that boost it, such as hypoxia, can be beneficial in treating cancer.

**Figure 10:**
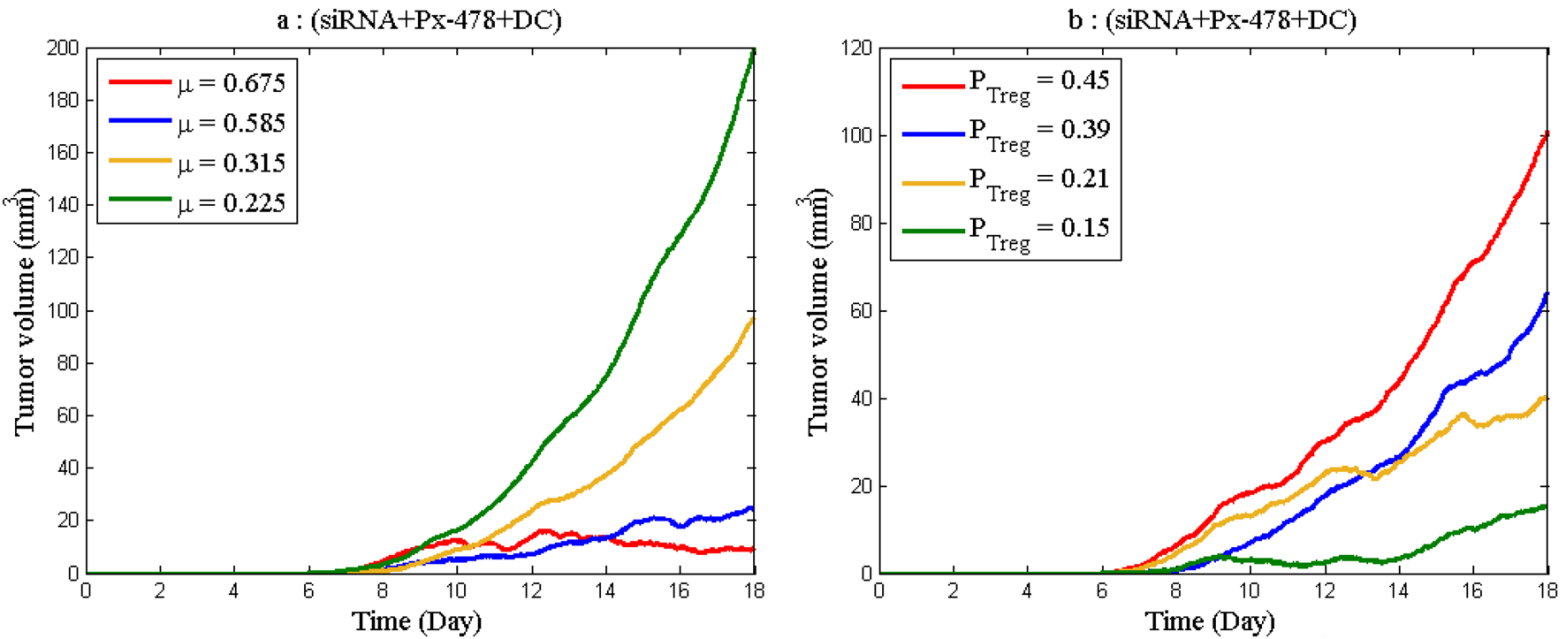
The time course of tumor volume as the parameters *µ* and *P*_*Treg*_ change. (**a**) Tumor volume as a result of the change in *µ*. (**b**) Tumor volume as a result of the change in *P*_*Treg*_.

The sensitivity of parameters *P*_*Treg*_ and *µ* are also high. For further investigation, the tumor volume has been examined in connection to changes in these parameters in Fig. 10. According to the results of the figure, it is necessary to identify factors that affect the production and cytotoxicity rate of effector cells. Increasing the cytotoxicity rate of the effector cells and their number leads to a decrease in tumor volume, and according to Fig. 5, decreasing the tumor volume reduces hypoxia, and according to Fig. 6, the final level of adenosine will decrease.

### Identifiability analysis

Uncorrelated parameters may be regarded as the crucial adjusting knobs of a model^41^. Interdependence among parameters makes the model unidentifiable, which means their values cannot be determined uniquely with the information at hand. Thus, characterizing the subset(s) of identifiable parameters and how they interact with each other and model output is crucial for a model. To perform this analysis, as explained in Ref.^41^, correlation coefficients between pairs of parameters were computed for different outputs in the model (see the Identifiability Analysis Method provided in the Appendices). Tumor volume, adenosine, hypoxia, effector cell cytotoxicity rate, number of effector cells, Treg cells, and DCs were considered as model outputs. If the correlation coefficient of a pair of parameters was equal to or greater than 0.9 in all outputs^42^, the corresponding parameters were interdependent and one of them could be eliminated from the model. According to the results of this analysis (Fig. A5 in Appendices), there is no pair of parameters in the model whose correlation coefficient is greater than or equal to 0.9 in all output, so, all the parameters in the model can be identified.

## Discussion

Activities to develop new cancer therapies have accelerated during the past several decades. Cancer immunotherapy is one of these developments and has significantly increased the survival chances of patients^43^. Mathematical modeling has been beneficial in improving our understanding of cancer and its various treatment methods. In this study, a spatio-temporal model for tumor growth and its interaction with the immune system has been presented. Also, hypoxia and adenosine were added to this model and their effects on TME and each other were examined. The model parameters were estimated using experimental data obtained from two distinct studies, investigating the effect of adenosine and hypoxia on DC-based immunotherapy, performed on 4T1 breast cancer-bearing mice. This model was used to investigate an effective treatment pattern. Model simulations predict that by applying this pattern, the tumor volume will decrease and the immune system will perform better than other groups.

The simulation results of this work show that adenosine grows strongly when hypoxia increases in the TME, and inhibiting hypoxia leads to a decrease in adenosine levels. Also, experimental studies confirm the adenosine increase due to the oxygen reduction^13,16^. In addition, in these studies, it has been shown that the increase in hypoxia leads to an increase in suppressor cells. Therefore, it can be concluded that an increase in hypoxia leads to an increase in adenosine, and according to Fig. 10, the number of suppressor cells increases with an increase in adenosine. As a result, hypoxia leads to the increase of suppressor cells both directly and indirectly through adenosine. Also, using sensitivity analysis, it was confirmed that adenosine has the greatest effect on tumor growth in TME compared to other model factors. Therefore, to achieve better therapeutic results, adenosine must be inhibited, either directly (with its inhibitory vaccine) or indirectly (for example, by inhibiting hypoxia).

Our model showed that the sooner the vaccine is injected, the more effective it is and the less the tumor grows. In some studies, it has been reported that a high tumor load may favor the initial expansion of immune cells, but on the other hand, it may also enhance the suppression of tumor immunity^44^. Thus, the optimal approach to using immunotherapy in conjunction with surgical resections is a question that can be addressed using model simulation with different scenarios for initial tumor size and immune system cells.

One of the challenges in vaccine-based treatments is finding the optimal dose and interval between injections. We used the proposed model to determine these factors. The results showed that increasing the dose or the number of injections does not always improve the treatment. For the DC vaccine, it was seen that there is a certain threshold beyond which increasing the vaccine dose not only does not reduce tumor growth but even increases it. This is because with increasing the number of injected DCs, the space around the tumor is completely occupied by DCs, preventing effector cells from getting to the tumor cells. Some studies also emphasize that in order to prevent the traffic of DCs and achieve successful treatment in DC immunotherapy, the appropriate and optimal number of DCs should be used^23,25^. In addition to the number, the location of the DCs and how they move in the network is important. Because the closer the DCs are to the T lymphocytes, the more likely the T lymphocytes will be activated, and this will improve cancer treatment. As a result, the number of DC cells, their locations in the grid, and their potential to activate T lymphocytes can all affect the tumor volume. By using spatial models, we have gained insights into the behavior of DC cells that temporal models, such as ODEs, have been unable to capture. This underscores the importance of incorporating spatial dynamics into our modeling approach in order to achieve a more comprehensive understanding of cellular phenomena.

In vaccination with inhibitors of hypoxia and adenosine, tumor growth is slowed by increasing the injected dose or the number of injections or reducing the time interval between injections. Adenosine inhibitory vaccine has more effect on the final volume of the tumor than the hypoxia inhibitory vaccine. The model was used to evaluate several injection strategies and identify the near-optimal injection pattern. For the inhibition of adenosine and hypoxia, three near-optimal strategies were found using a trial-and-error manner (indicated with ★ symbols in Table 3).

The sensitivity analysis of the model showed that the first three sensitive parameters of the model are *β*, *r*_*At*_, and *r*, which are all related to the adenosine dynamic. According to all the mentioned results, it can be concluded that adenosine is more involved in the tumor growth than hypoxia. Another study has also emphasized that among suppressive factors, it seems that adenosine exerts higher immunosuppressive function as its producing molecules including CD73 are expressed on both tumor and immune cells^3^. Additionally, *µ* and *P*_*Treg*_ (effector cell cytotoxicity rate and Treg production rate, respectively) are also important parameters, which are also emphasized in other studies^25^. Targeting these parameters can be considered in designing treatment strategies.

We can mention several limitations for the presented model to be considered in future works. The parameters of this model are estimated for a time frame of 18 days. Measuring tumor size over a longer period of time can better show the effect of hypoxia on the tumor. Because as the tumor grows, its metabolism elevates and the need for oxygen increases, which causes hypoxia. Investigating the model in larger time frames will force us to consider other phenomena such as angiogenesis (something that is usually not seen in the early days of tumor formation). Formation of blood vessels near the tumor can improve oxygen delivery to it and have a direct effect on hypoxia dynamics. In this work, we considered the effect of hypoxia on adenosine, but the effect of adenosine on hypoxia was not investigated. The feedback that these two factors have on each other can be a determining factor in the dynamics observed in the experimental data. In the proposed model, we considered that the tumor has a single layer, and therefore, all tumor cells consume oxygen similarly. The tumor can be considered as a three layers mass (as is considered in Ref.^22^), in which the inner layer is necrotic, the middle layer is quiescent and the outer layer is proliferating. These three layers have different oxygen consumption rates, and by considering them, more accurate dynamics of oxygen concentration in the TME can be obtained. We have endeavored to estimate the maximum number of parameters using our experimental data sourced from Refs.^3,4^. Notably, the data from both^3,4^ were derived from the same mouse models and tumor type, rendering them highly compatible. For parameters not directly available from these references, reasonable initial values were gleaned from other studies and fine-tuned within the model. It is conceivable that with more extensive experimental data, all model parameters could be estimated from a single dataset, thereby reducing uncertainty within the model. Finally, expanding the model and adding details of the tumor growth, cancer-promoting factors, and its interaction with other immune cells can help our understanding of the development and progression of the tumor and the use of targeted treatments, which will lead to improving current immunotherapies.

## Conclusions

Overall, in this study, we presented a spatio-temporal model to investigate the role of hypoxia, adenosine, and DC cells in cancer. The simulations showed that these factors affect the proliferation of cancer and hypoxia also affects the level of adenosine. We also concluded that adenosine has a greater effect than hypoxia in TME. Also, the simulations showed that the efficacy of DC-based immunotherapy is strongly dependent on the injection rate and the trafficking of DCs in TME. In addition, we introduced near-optimal treatment protocols and showed that the tumor volume is significantly reduced with these protocols.

### Supplementary Information

Supplementary Information.

## Data Availability

The code pertaining to siRNA + Px-478 + DC group is available on GitHub at: https://github.com/elaheghiyabi96/Modeling-Tumor-Escape-Mechanisms-for-DC-Based-Immunotherapy-Insights-into-the-Role-of-Hypoxia-and-A. The datasets used and analyzed the current study are available from the corresponding author upon reasonable request.
