# Supplementary material for: Mathematical modeling of hypoxia and adenosine to explore tumor escape mechanisms in DC-based immunotherapy
Source: Sci Rep. 2024 May 18;14:11387. doi: 10.1038/s41598-024-62209-6 (PMC11102449; doi:10.1038/s41598-024-62209-6)
Supplement: Supplementary file 1 — Supplementary Information. [file 41598_2024_62209_MOESM1_ESM.docx]

**Supplementary Material:**

**Mathematical Modeling of Hypoxia and Adenosine to Explore Tumor Escape Mechanisms in DC-Based Immunotherapy**

Elahe Ghiyabi^a^, Abazar Arabameri^b*^, Mostafa Charmi^c^

^a^ Department of Electrical Engineering, University of Zanjan, Zanjan, Iran, elaheghiyabi1996@gmail.com

^b^ Department of Electrical Engineering, University of Zanjan, Zanjan, Iran, arabameri@znu.ac.ir

^c^ Department of Electrical Engineering, University of Zanjan, Zanjan, Iran, charmi.mostafa@znu.ac.ir

**Appendices**

**Appendix A: Supplementary Figures**


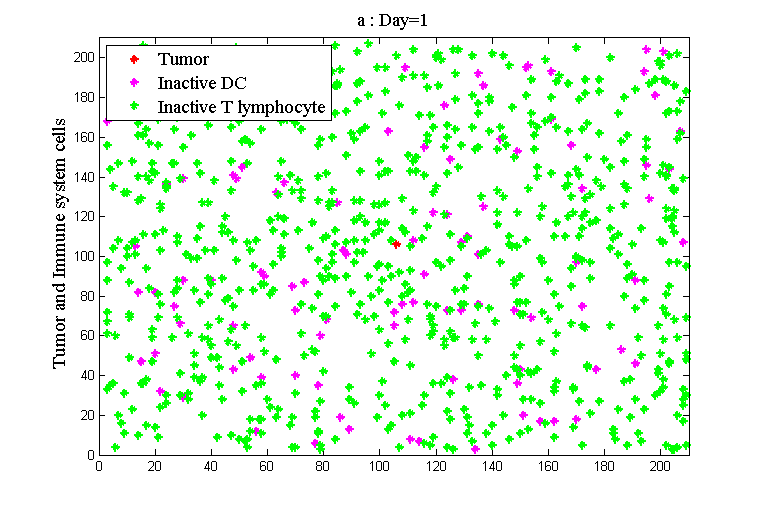

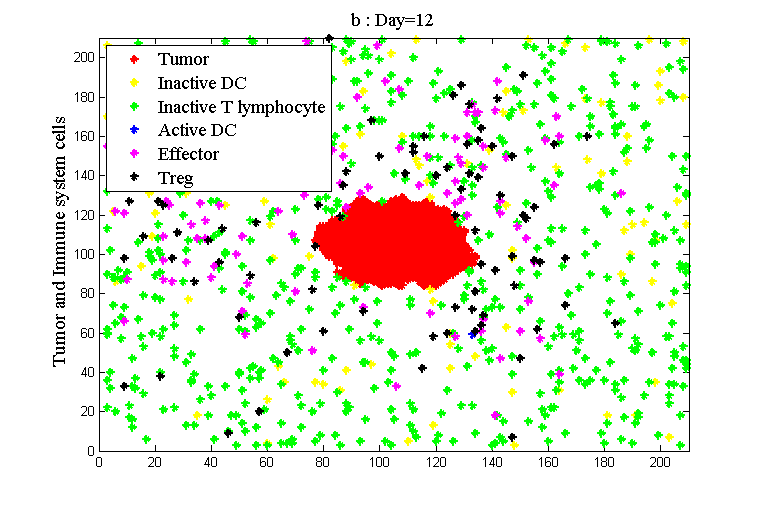


**Figure A1.** A representation of the model lattice containing tumor and the immune cells. (a) On day 1, the tumor cell is in the center of the grid and the immune cells are inactive and randomly distributed throughout the grid. (b) On day 12, the tumor cell has grown, immune cells have activated and moved, and occupied different positions in the grid.


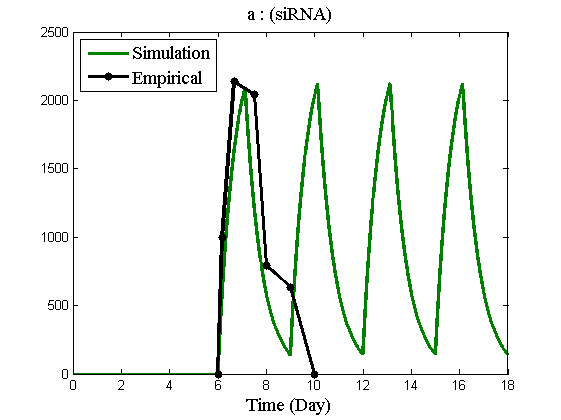

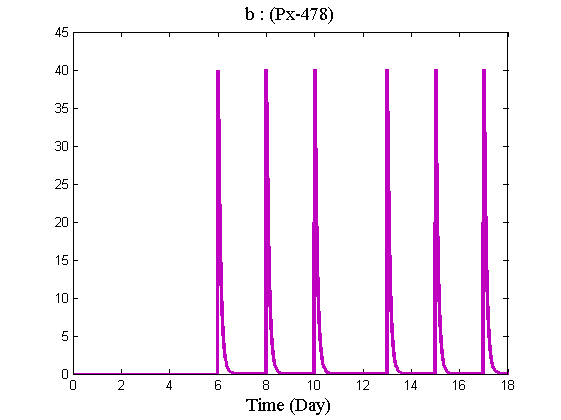


**Figure A2.** The time course of adenosine and hypoxia inhibitory vaccines. (a) siRNA vaccine up to 18 days and is injected on days 6, 9, 12, and 15. (b) Px-478 vaccine up to 18 days and is injected on days 6, 8, 10, 13, 15, and 17.


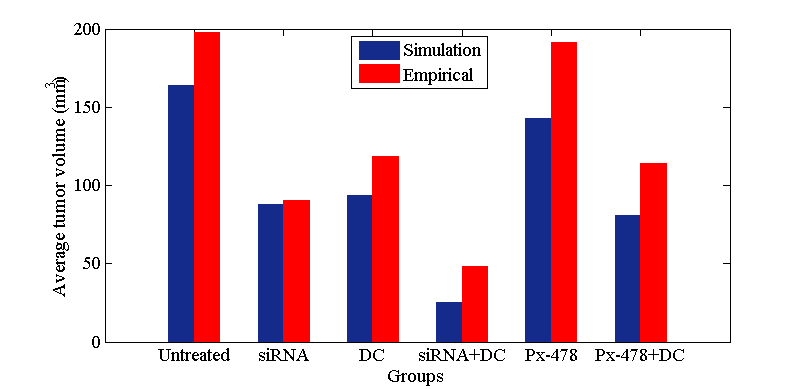


**Figure A3.** Average tumor volume for the empirical data ^3,4^ and model for the present study output in different groups. The results show that the model can greatly show the patterns and behavior of the experimental data.


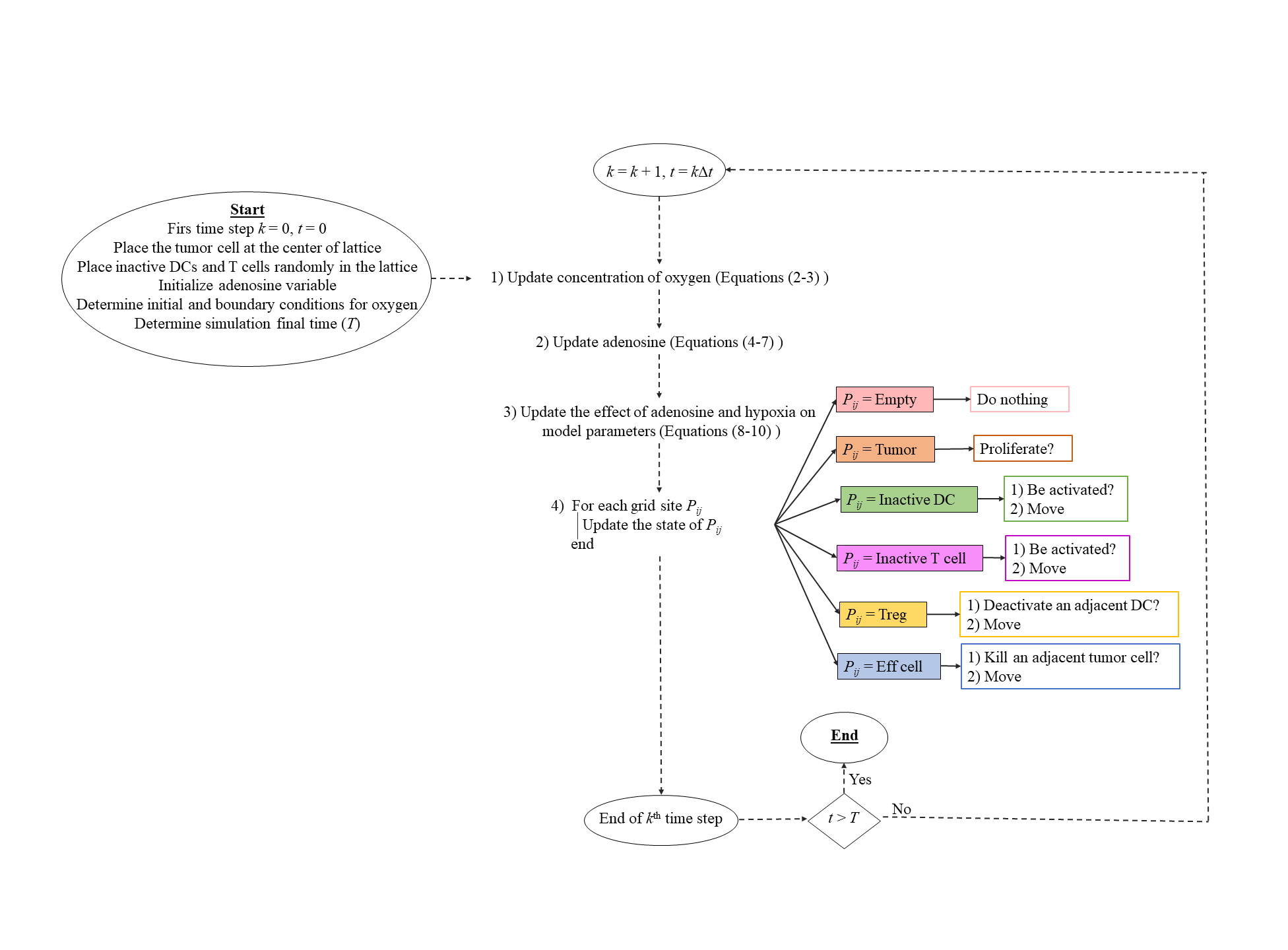


**Figure A4.** A graphical representation of the sequential steps involved in simulating the proposed model**.**

**Global sensitivity analysis**

For sensitivity analysis, the method described in ^45^ was employed. Initially, a parameter range was defined for each model parameter, with lower and upper limits set at 70% and 130% of the respective values listed in Table 1. Subsequently, 200 equidistant points were selected within this interval and randomly arranged in a column. These columns, representing different parameters, were then concatenated to form an *n* × *m* matrix called *X_P_* (where *n* = 200 and *m* denotes the number of model parameters). Each row in this matrix represented a distinct parameter set for the model. By inputting these parameter sets into the model, along with the initial conditions explained in section 3.1, the model was simulated for a period of 18 days, and the average tumor size over this period was considered as the output for that particular row. Consequently, a *Y_P_* vector of size 200 × 1 was obtained. The sensitivity value of the model to each parameter was determined by calculating the correlation coefficient between each column of *X_P_* and *Y_P_*, yielding a sensitivity value ranging from -1 to 1 for the corresponding parameter. For further details, please refer to Figure 1 in ^45^.

**Identifiability analysis**

To conduct this analysis, we employed a method similar to the one described in ^46^. The approach involved considering the tumor size, adenosine, hypoxia, effector cell cytotoxicity rate, number of effector cells, Treg cells, and DCs as the output over an 18-day period (with initial conditions outlined in section 3.1). Numerical differentiation was then applied to calculate the derivative of the output with respect to the *i*th parameter of the model. Specifically, the output was computed twice: once using 1.15*p_i_* as the *i*th parameter value and again using 0.85*p_i_*, while keeping the other parameters fixed at the values provided in Table 1 (where *p_i_* represents the value of the *i*th parameter in Table 1). This process resulted in a derivative vector, denoted as *d_i_* = Δ*y*/Δ*p_i_*, where Δ*y* represents the change in the output and Δ*p_i_* represents the change in the *i*th parameter. Subsequently, the correlation coefficient between different *d_i_* values was calculated, leading to the creation of a *m*×*m* matrix, denoted as *C* (where *m* represents the number of parameters). If the absolute value of the correlation coefficient (|*c*_ij_|) exceeded a predefined threshold ξ (set at ξ=0.9), it indicated a strong correlation between the parameter pair.


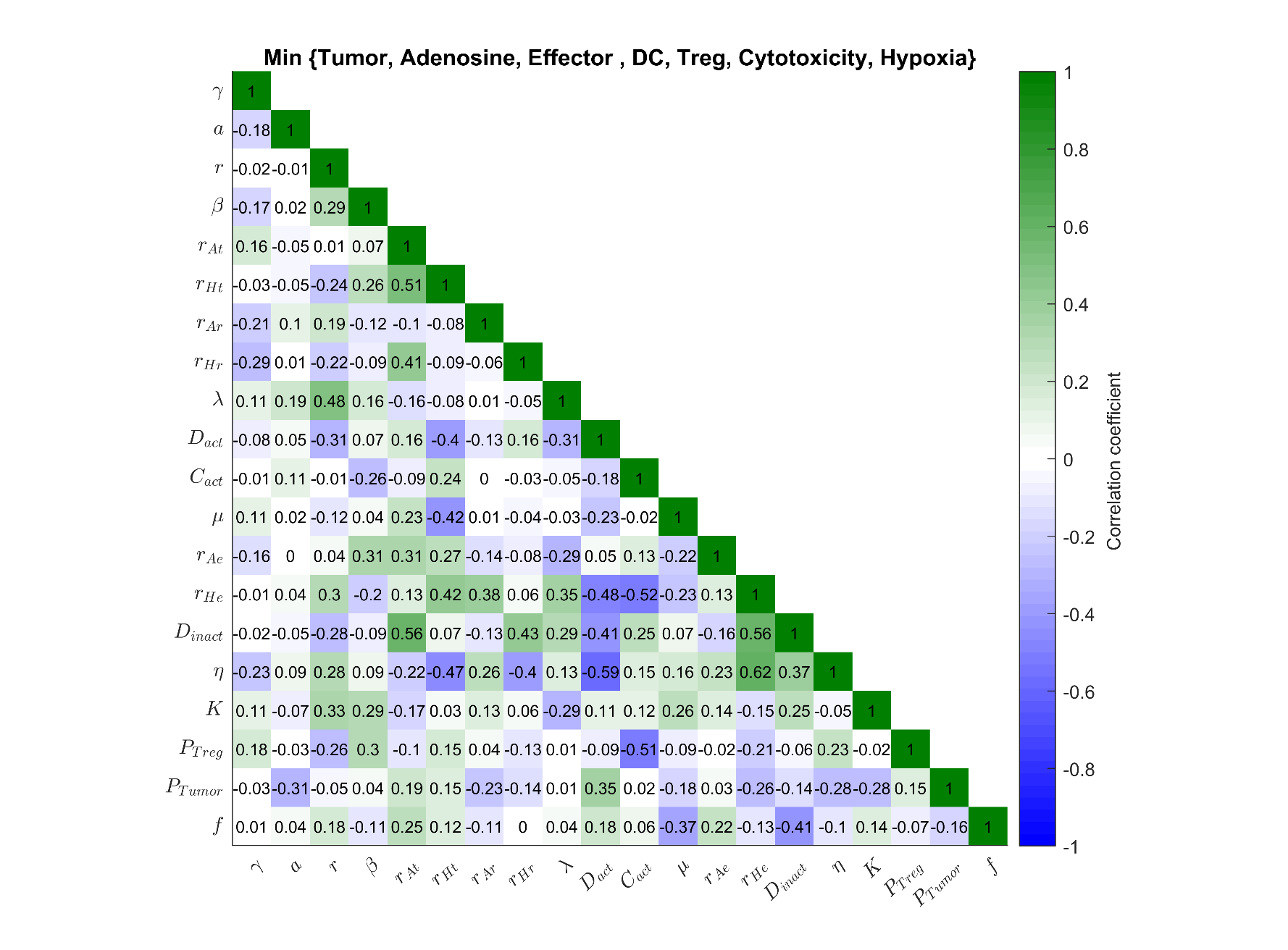


**Figure A5.** Parameter correlation matrix. Each component of the matrix is the minimum of correlation coefficients between the sensitivities of a parameter for different outputs (tumor, adenosine, effector cell, DC, Treg, cytotoxicity, and hypoxia).

**References**

1 Bray, F. *et al.* Global cancer statistics 2018: GLOBOCAN estimates of incidence and mortality worldwide for 36 cancers in 185 countries. *CA: a cancer journal for clinicians* **68**, 394-424 (2018).

2 Mattiuzzi, C. & Lippi, G. Current cancer epidemiology. *Journal of epidemiology and global health* **9**, 217 (2019).

3 Jadidi-Niaragh, F. *et al.* CD73 specific siRNA loaded chitosan lactate nanoparticles potentiate the antitumor effect of a dendritic cell vaccine in 4T1 breast cancer bearing mice. *Journal of Controlled Release* **246**, 46-59 (2017).

4 Kheshtchin, N. *et al.* Inhibition of HIF-1α enhances anti-tumor effects of dendritic cell-based vaccination in a mouse model of breast cancer. *Cancer Immunology, Immunotherapy* **65**, 1159-1167 (2016).

5 Muz, B., de la Puente, P., Azab, F. & Kareem Azab, A. The role of hypoxia in cancer progression, angiogenesis, metastasis, and resistance to therapy. *Hypoxia*, 83-92 (2015).

6 Noman, M. Z. *et al.* Hypoxia: a key player in antitumor immune response. A review in the theme: cellular responses to hypoxia. *American Journal of Physiology-Cell Physiology* **309**, C569-C579 (2015).

7 Emami Nejad, A. *et al.* The role of hypoxia in the tumor microenvironment and development of cancer stem cell: a novel approach to developing treatment. *Cancer Cell International* **21**, 1-26 (2021).

8 Mortezaee, K. & Majidpoor, J. The impact of hypoxia on immune state in cancer. *Life sciences* **286**, 120057 (2021).

9 Antonioli, L., Pacher, P., Vizi, E. S. & Haskó, G. CD39 and CD73 in immunity and inflammation. *Trends in molecular medicine* **19**, 355-367 (2013).

10 Allard, B. *et al.* Anti‐CD73 therapy impairs tumor angiogenesis. *International journal of cancer* **134**, 1466-1473 (2014).

11 Arab, S. & Hadjati, J. Adenosine blockage in tumor microenvironment and improvement of cancer immunotherapy. *Immune Network* **19** (2019).

12 Morandi, F., Horenstein, A., Rizzo, R. & Malavasi, F. The role of extracellular adenosine generation in the development of autoimmune diseases. *Mediators of inflammation* **2018** (2018).

13 Conde, S. V. & Monteiro, E. C. Hypoxia induces adenosine release from the rat carotid body. *Journal of neurochemistry* **89**, 1148-1156 (2004).

14 Goueli, S. A. & Hsiao, K. Monitoring and characterizing soluble and membrane-bound ectonucleotidases CD73 and CD39. *PloS one* **14**, e0220094 (2019).

15 Hatfield, S. M. *et al.* Systemic oxygenation weakens the hypoxia and hypoxia inducible factor 1α-dependent and extracellular adenosine-mediated tumor protection. *Journal of molecular medicine* **92**, 1283-1292 (2014).

16 Hatfield, S. M. *et al.* Immunological mechanisms of the antitumor effects of supplemental oxygenation. *Science translational medicine* **7**, 277ra230-277ra230 (2015).

17 Bell, D., Young, J. W. & Banchereau, J. Dendritic cells. *Advances in immunology* **72**, 255-324 (1999).

18 Mellman, I. Dendritic cells: master regulators of the immune response. *Cancer immunology research* **1**, 145-149 (2013).

19 Schraml, B. U. & e Sousa, C. R. Defining dendritic cells. *Current opinion in immunology* **32**, 13-20 (2015).

20 Veglia, F. & Gabrilovich, D. I. Dendritic cells in cancer: the role revisited. *Current opinion in immunology* **45**, 43-51 (2017).

21 Gammon, K. Mathematical modelling: Forecasting cancer. *Nature* **491**, S66-S67 (2012).

22 Pourhasanzade, F. & Sabzpoushan, S. A new mathematical model for controlling tumor growth based on microenvironment acidity and oxygen concentration. *BioMed Research International* **2021** (2021).

23 Arabameri, A. & Pourgholaminejad, A. Modeling codelivery of CD73 inhibitor and dendritic cell-based vaccines in cancer immunotherapy. *Computational Biology and Chemistry* **95**, 107585 (2021).

24 Chimal-Eguia, J. C., Castillo-Montiel, E., Rangel-Reyes, J. C. & Paez-Hernández, R. T. Modeling Dendritic Cell Pulsed Immunotherapy for Mice with Melanoma—Protocols for Success and Recurrence. *Applied Sciences* **11**, 3199 (2021).

25 Macfarlane, F. R., Lorenzi, T. & Chaplain, M. A. Modelling the immune response to cancer: an individual-based approach accounting for the difference in movement between inactive and activated T cells. *Bulletin of mathematical biology* **80**, 1539-1562 (2018).

26 Pourhasanzade, F., Sabzpoushan, S., Alizadeh, A. M. & Esmati, E. An agent-based model of avascular tumor growth: Immune response tendency to prevent cancer development. *Simulation* **93**, 641-657 (2017).

27 Robertson-Tessi, M., El-Kareh, A. & Goriely, A. A mathematical model of tumor–immune interactions. *Journal of theoretical biology* **294**, 56-73 (2012).

28 Arabameri, A. & Arab, S. Understanding the Interplay of CAR-NK Cells and Triple-Negative Breast Cancer: Insights from Computational Modeling. *Bulletin of Mathematical Biology* **86**, 20 (2024).

29 Xie, Z.-X. *et al.* Role of the immunogenic and tolerogenic subsets of dendritic cells in multiple sclerosis. *Mediators of inflammation* **2015** (2015).

30 Bhat, R. & Watzl, C. Serial killing of tumor cells by human natural killer cells–enhancement by therapeutic antibodies. *PloS one* **2**, e326 (2007).

31 Hirota, K. Basic biology of hypoxic responses mediated by the transcription factor HIFs and its implication for medicine. *Biomedicines* **8**, 32 (2020).

32 Liu, Y. J. *et al.* Research progress on adenosine in central nervous system diseases. *CNS neuroscience & therapeutics* **25**, 899-910 (2019).

33 Liu, H. & Xia, Y. Beneficial and detrimental role of adenosine signaling in diseases and therapy. *Journal of applied physiology* **119**, 1173-1182 (2015).

34 Patente, T. A. *et al.* Human dendritic cells: their heterogeneity and clinical application potential in cancer immunotherapy. *Frontiers in immunology* **9**, 3176 (2019).

35 Christophe, C. *et al.* A biased competition theory of cytotoxic T lymphocyte interaction with tumor nodules. *PloS one* **10**, e0120053 (2015).

36 Goya, G. *et al.* Dendritic cell uptake of iron-based magnetic nanoparticles. *Cell biology international* **32**, 1001-1005 (2008).

37 Rozenberg, G. *Microscopic haematology: a practical guide for the laboratory*. (Elsevier Australia, 2011).

38 Aghighi, M. *et al.* Three-dimensional radiologic assessment of chemotherapy response in Ewing sarcoma can be used to predict clinical outcome. *Radiology* **280**, 905-915 (2016).

39 Su, L. F., Del Alcazar, D., Stelekati, E., Wherry, E. J. & Davis, M. M. Antigen exposure shapes the ratio between antigen-specific Tregs and conventional T cells in human peripheral blood. *Proceedings of the National Academy of Sciences* **113**, E6192-E6198 (2016).

40 Li, X. *et al.* Induction of type 2 T helper cell allergen tolerance by IL-10–differentiated regulatory dendritic cells. *American journal of respiratory cell and molecular biology* **42**, 190-199 (2010).

41 Gábor, A., Villaverde, A. F. & Banga, J. R. Parameter identifiability analysis and visualization in large-scale kinetic models of biosystems. *BMC systems biology* **11**, 1-16 (2017).

42 Kimmel, G. J., Locke, F. L. & Altrock, P. M. The roles of T cell competition and stochastic extinction events in chimeric antigen receptor T cell therapy. *Proceedings of the Royal Society B* **288**, 20210229 (2021).

43 Esfahani, K. *et al.* A review of cancer immunotherapy: from the past, to the present, to the future. *Current Oncology* **27**, 87-97 (2020).

44 León-Triana, O., Pérez-Martínez, A., Ramírez-Orellana, M. & Pérez-García, V. M. Dual-target CAR-Ts with on-and off-tumour activity may override immune suppression in solid cancers: A mathematical proof of concept. *Cancers* **13**, 703 (2021).

45 Marino, S., Hogue, I. B., Ray, C. J. & Kirschner, D. E. A methodology for performing global uncertainty and sensitivity analysis in systems biology. *Journal of theoretical biology* **254**, 178-196 (2008).

46 Brady-Nicholls, R. *et al.* Prostate-specific antigen dynamics predict individual responses to intermittent androgen deprivation. *Nature communications* **11**, 1750 (2020).
